# Automated red cell exchange in acute sickle cell crises: Case series and review of the literature

**DOI:** 10.1016/j.htct.2026.106454

**Published:** 2026-04-22

**Authors:** V. Aishwarya, Soumya Das, Juilee Shalik Charmode, Parag Fulzele, Rounak Dubey, Neema Vijay

**Affiliations:** Department of Transfusion Medicine, All India Institute of Medical Sciences, Nagpur, Maharashtra, India

**Keywords:** Sickle cell disease, Erythrocytapheresis, Red cell exchange, Vaso-occlusive crisis, Acute chest syndrome

## Abstract

Automated red cell exchange (erythrocytapheresis) offers rapid suppression of circulating hemoglobin S without adding iron burden and is increasingly used to escalate care in acute sickle cell disease complications. This study describes four patients with severe vaso-occlusive crisis and/or acute chest syndrome managed with automated red cell exchange in a tertiary-care intensive care unit in India and summarizes Indian reports on its feasibility and on early outcomes. In the Indian scenario, this therapy is underutilized mostly due to the limited-resource setting, non-affordability, and probable lack of awareness of its availability.

## Introduction

Sickle cell disease (SCD) is an autosomal-recessive disorder of the *β-globin* gene that substitutes glutamic acid with valine at codon 6 leading to the production of hemoglobin S (Hb S), which under deoxygenated conditions undergoes polymerization and consequent sickling [1,2]. This change promotes endothelial adhesion, microvascular obstruction, tissue ischemia/infarction, and hemolysis [1,3].

The clinical spectrum of the disease spans acute and chronic complications. Common acute events include painful vaso-occlusive crises (VOC), acute chest syndrome (ACS), stroke, and hepatic or splenic sequestration, while longer-term morbidity reflects cumulative organ damage and growth impairment. Of these complications, VOC remain the clinical hallmark and the most frequent reason for hospitalization [4].

Red blood cell transfusion remains a core supportive therapy aimed at improving oxygen-carrying capacity and reducing circulating Hb S. Depending on indication, simple transfusion is used to raise hemoglobin levels however, automated red cell exchange (RCE; erythrocytapheresis) is preferred when rapid Hb S reduction is required for high-risk complications such as acute stroke or severe ACS [5, 6, 7].

Automated RCE offers significant advantages in this regard, as it rapidly reduces the percentage of Hb S, decreases blood viscosity, and improves oxygen delivery without causing excessive iron accumulation [7].

This study presents a case series of sickle cell crises managed with RCE in an intensive care unit (ICU) setting in a tertiary care center. This study also reviewed the literature to assess the utilization and clinical efficiency of this therapy in India.

## Case 1

A 9-year-old male patient with homozygous SCD presented with severe limb and back pain and a past history of right hemiparesis and inconsistent hydroxyurea. Baseline Hb S was 80.4%. Automated RCE was performed using a continuous-flow system via a left femoral double-lumen catheter; the circuit was primed with packed red blood cells (PRBC). A total volume of 775 mL of leukoreduced PRBCs was exchanged at an inlet flow rate of 10 mL/min. The procedure targeted a final Hb S concentration of ≤30% and a hematocrit (Hct) of approximately 30%. Continuous IV calcium was given. The procedure was uneventful; pain and mobility improved markedly and Hb S fell to 16.6%. He was discharged on hydroxyurea, folate, and calcium. The patient remained ambulatory (able to walk independently) and clinically stable at the time of follow-up.

## Case 2

A 26-year-old male patient presenting with VOC and ACS exhibited chest pain, diffuse aches, and a transient altered sensorium. Following initial supportive therapy, automated RCE was initiated due to deteriorating clinical symptoms. The procedure was performed via right femoral access, exchanging 2403 mL of PRBCs at an inlet flow rate of 30 mL/min. The procedure targeted a Hb S level of ≤30% and a Hct of approximately 25%, with concurrent continuous IV calcium supplementation. Following the exchange, the patient’s respiratory status and mentation improved significantly; his Hb S level decreased from 64.6% to 25.1% within 24 h. He was subsequently discharged on hydroxyurea and nutritional supplements

## Case 3

A 19-year-old female patient with prolonged VOC and ACS was admitted to the ICU with severe multiorgan failure, including acute respiratory distress. Automated RCE, performed via right femoral access, exchanged 905 mL PRBCs at an inlet rate of 30 mL/min. The procedure targeted a Hb S level of ≤30% and a Hct of approximately 25%, with concurrent continuous IV calcium supplementation. Twenty-four hours after the exchange, the Hb S level had dropped from 62.7% to 6.7%. Despite this laboratory response, there was no immediate clinical improvement, and intensive support was continued.

## Case 4

A 16-year-old male patient with a history of intermittent hydroxyurea adherence presented with pleuritic chest pain, severe bone pain, fever, and hypoxemia. Chest radiography revealed new basilar opacities consistent with **ACS**. Due to persistent hypoxemia, an automated RCE was performed via right internal jugular access. A total of 1800 mL of PRBCs was exchanged, targeting a Hb S level of ≤30% and a Hct of approximately 25–30%, with concurrent IV calcium supplementation. Post-procedure chills were managed symptomatically. Clinical recovery was rapid, characterized by reduced oxygen requirements and the abatement of chest pain. The Hb S level decreased from 74.5% to 24.1% within 24 h, and the patient was discharged with an incentive spirometry regimen.

The pre- and post-automated RCE hematological parameters and clinical outcomes for all four cases are summarized in Table 1.

**Table 1:** Hematological parameters before and after automated red cell exchange and clinical outcome.

Case	Indication	Exchange volume (mL)		Pre-procedure (%)	Post-procedure (%)	Clinical outcome
1	Severe VOC, high Hb S	775	Hb S	80.4 g/dL	16.6	Rapid pain relief; ambulatory at follow-up
			Hb	7.7 g/dL	10.7	
			Hct	22.9%	32	
2	VOC + ACS; altered sensorium	2403	Hb S	64.6 g/dL	25.1	Improved oxygenation and mentation
			Hb	10.9 g/dL	7.6	
			Hct	31.0%	26	
3	VOC + ACS with respiratory failure	905	Hb S	62.7 g/dL	6.7	No immediate clinical improvement
			Hb	8.7 g/dL	7.8	
			Hct	26.4%	24.7	
4	ACS with hypoxemia	1800	Hb S	74.5 g/dL	24.1	Rapid improvement; discharged with spirometry
			Hb	8.2 g/dL	8.3	
			Hct	22.5%	23.8	

VOC: Vaso-occlusive crisis; ACS: Acute chest syndrome; Hct: hematocrit, Hb: hemoglobin.

## Discussion

In this four-patient case series, automated RCE was instituted for severe VOC or ACS refractory to initial supportive measures. Procedurally, all automated RCEs were performed using the Spectra Optia Apheresis System (Terumo BCT) on a continuous-flow platform. The procedures utilized leukoreduced, cross-match-compatible PRBCs, with prophylactic IV calcium administered throughout. Post-exchange targets were a hematocrit of approximately 25–30% and Hb S ≤ 30%; in all cases, Hb S decreased to target levels. Three patients demonstrated prompt clinical improvement, while one intensive-care patient with advanced ACS had laboratory response but no immediate clinical response as automated RCE was performed at an advanced stage and the patient had already severe preexisting multiorgan failure. No serious procedure-related adverse events occurred in all four patients.

Automated RCE is strongly recommended for acute sickle cell complications such as stroke, severe or rapidly progressive ACS with hypoxia, acute multiorgan failure, severe sickle cell hepatopathy, prolonged ischemic priapism, preoperative optimization in moderate to high-risk surgeries, and rapidly evolving severe symptoms [7, 8, 9].

As per the American Society for Apheresis (ASFA) guidelines, automated RCE in SCD is used in acute and nonacute conditions [10]. However, the category of indication and Grade of recommendation varies with each condition as listed in Table 2.

**Table 2:** Category and grade of recommendation for red cell exchange in sickle cell disease (SCD).

SCD	Indication	Category	Grade
Acute	Acute stroke	I	1C
	Acute chest syndrome, severe	II	1C
	Other complication	III	2C
Non-acute	Stroke prophylaxis	I	1A
	Pregnancy	II	2B
	Recurrent vaso-occlusive pain crisis	II	2B
	Preoperative management	III	2A

Adopted from American Society for Apheresis Guidelines [10].

## Review of literature

Published Indian data dedicated to automated RCE for VOC are limited and largely consist of single-centre experiences where VOC represents a minority of indications for acute crises. The Rapid Preferred Reporting Items for Systematic reviews and Meta-Analysis (PRISMA) guidelines were used for this review. India-based primary studies (case reports/series or cohorts) that used automated RCE for VOC or mixed acute crises that reported at least one outcome (change in Hb S percentage, clinical response, or adverse events) were included. Non-India populations and non-automated RCE interventions were excluded.

**Figure 1:**
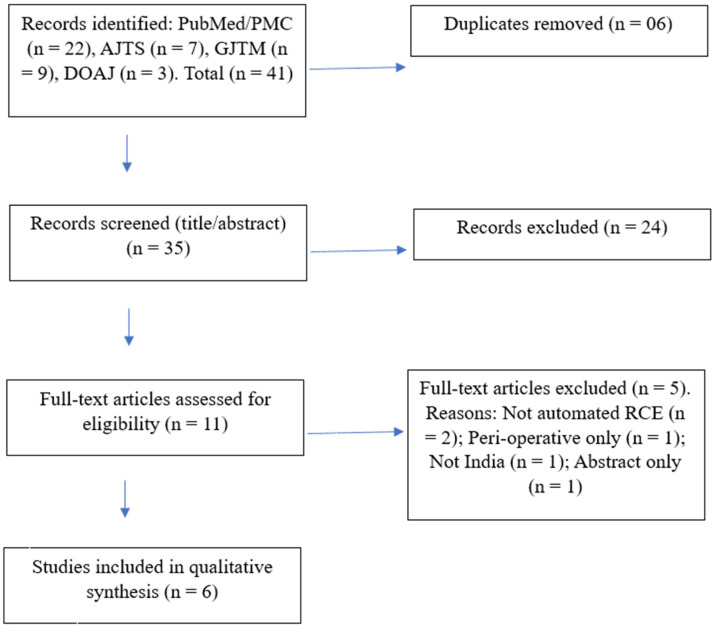
PRISMA chart showing A summary of data extraction. AJTS: Asian Journal of Transfusion Science; GJTM: Global Journal of Transfusion Medicine; RCE: Red cell exchange.

PubMed/PMC and Indian transfusion journals (Asian Journal of Transfusion Science; Global Journal of Transfusion Medicine/Medknow; DOAJ) were searched using combinations of ‘India’, ‘automated red cell exchange’, ‘erythrocytapheresis’, ‘sickle cell’, ‘vaso-occlusive crisis’, and ‘acute chest syndrome’ with the titles and abstracts being screened for India-based automated RCE in SCD. Full texts were reviewed for automated RCE and acute indication as shown in Figure 1.

The peri‑operative cohort by Daniel et al., although not focused on VOC, included 21 automated RCE procedures in 18 SCD patients undergoing surgery for avascular necrosis. Hb S was reduced from 73 to 85% to 22–29%, thus meeting transfusion targets with minimal adverse reactions. While the indication was surgical optimization, the findings support the technical feasibility and safety of automated RCE in India [11]. The five-patient series (eight exchanges) of Chowdhry et al. included patients with mixed acute crises. VOC-specific data were not separated. A target fractional cell residual of ≤30% was consistently achieved. The presence of alloantibodies in two patients underscored the significant challenges for blood supplies in India [12]. Iyyapan et al. published a single case report of acute bone-pain VOC. After one automated RCE session, the Hb S was reduced from 74% to 18%, with rapid resolution of symptoms demonstrating clear clinical benefit of this procedure in cases of severe VOC [13]. Rajendran et al. managed a patient with VOC and ACS using two successive automated RCE sessions, effectively reducing the Hb S level to 16%. Following clinical recovery, the authors highlighted the underutilization of automated RCE in India, citing limited awareness, constrained resources, and poor donor availability [7]. Kanungo et al. reported a retrospective experience from Eastern India involving nine SCD patients who underwent automated RCE; among these, two presented with VOC. Mean post-procedure Hb S was approximately 30.7%, with no procedure-related complications reported. This demonstrates feasibility and safety of automated RCE even outside major metropolitan centres [14]. Zanzari et al. conducted a prospective observational study of eight patients with acute crises, including one with VOC and seven with ACS. Following automated RCE, Hb S levels fell significantly from 68.5% to 16.4% with parallel improvements observed in oxygenation and vital signs. This highlights the rapid physiological benefits produced by automated RCE [15]. Table 3 describes the various characteristics of the SCA cases in India managed by automated RCE.

**Table 3:** Characteristics of the sickle cell disease cases in India managed by automated red cell exchange.

Study (Year)	Design	VOC as indication	Other indications	Target Achieved	Outcomes / Safety
Daniel et al., [11] 2016	Retrospective; 21 exchanges (18 SCD)	No (pre-operative)	AVN optimization	Hb S: 22–29%	Targets met, minimal reactions
Chowdhry et al., [12] 2021	Retrospective series; n = 5 (8 exchanges)	Not separated (mixed acute crises)	Mixed; pre-op 1	FCR: ≤30%	Safe/effective
Iyyapan et al., [13] 2023	Case report; n = 1	Yes (acute bone pain)	—	Hb S: 74% → 18%	Prompt symptom relief
Rajendran et al., [7] 2023	Case report + review; n = 1	Painful crisis with ACS	ACS, hemolytic crisis	Hb S; 16% after 2 RCEs	Rapid clinical recovery under-utilization noted
Kanungo et al., [14] 2024	Retrospective series; n = 9	2 cases of 9	Pre-op 3/9; hepatopathy 2/9; others	Mean post-Hb S: ≈30.7%	Symptoms & lab improvement
Zanzari et al., [15] 2024	Prospective observational; n = 8	1/8 (ACS 7/8; sequestration 1/8)	ACS predominant	Hb S: 68.45% → 16.44%	Improved vitals

VOC: Vaso-occlusive crisis; ACS: Acute chest syndrome; AVN: Avascular necrosis; Hb: Hemoglobin; FCR: Fractional cell residual.

## Conclusion

Automated RCE is a promising acute treatment for indicated SCD complications. In the Indian context, this therapy remains underutilized, primarily due to resource constraints, high costs, a shortage of trained staff, and the requirement for large volumes of blood. Furthermore, limited awareness of its availability and the challenges of securing compatible units for alloimmunized patients remain significant barriers. Further studies should be conducted in India to explore the evidence of the efficacy of automated RCE in the acute management of SCA crises. Expanded prospective, multicentre studies are needed in India to refine patient selection and assess long-term outcomes.

## Funding

The author(s) received no specific funding for this work

**Data Availability Statement**

Not applicable

## Informed consent

A written informed consent obtained from the patient

## Author contribution

VA: writing -original draft, Conceptualization, Formal analysis

SD: writing-review/ and editing

JSC: writing-review/ and editing

RD: Supervision

PF: Supervision

NV: PData curation

## Conflicts of interest

None
